# A new species of *Munida* Leach, 1820 (Crustacea: Decapoda: Anomura: Munididae) from seamounts of the Nazca-Desventuradas Marine Park

**DOI:** 10.7717/peerj.10531

**Published:** 2021-01-05

**Authors:** María de los Ángeles Gallardo Salamanca, Enrique Macpherson, Jan M. Tapia Guerra, Cynthia M. Asorey, Javier Sellanes

**Affiliations:** 1 Sala de Colecciones Biológicas, Facultad de Ciencias del Mar, Universidad Católica del Norte, Coquimbo, Chile; 2 Departamento de Biología Marina & Núcleo Milenio Ecología y Manejo Sustentable de Islas Oceánicas, Universidad Católica del Norte, Larrondo 1281, Coquimbo, Chile; 3 Centre d’Estudis Avançats de Blanes (CEAB-CSIC), Blanes, Spain; 4 Programa de Magister en Ciencias del Mar Mención Recursos Costeros, Facultad de ciencias del Mar, Universidad Católica del Norte, Coquimbo, Chile

**Keywords:** Nazca ridges, Deep-sea, Pacific Ocean, Indo-pacific fauna, Salas & Gómez ridges

## Abstract

*Munida diritas* sp. nov. is described for the seamounts near Desventuradas Islands, in the intersection of the Salas & Gómez and Nazca Ridges, Chile. Specimens of the new species were collected in the summit (∼200 m depth) of one seamount and observed by ROV at two nearby ones. This species is characterized by the presence of distinct carinae on the thoracic sternites 6 and 7. Furthermore, it is not related with any species from the continental shelf nor the slope of America, while it is closely related to species of *Munida* from French Polynesia and the West-Pacific Ocean (i.e., *M. ommata, M. psylla* and *M. rufiantennulata*). In situ observations indicate that the species lives among the tentacles of ceriantarid anemones and preys on small crustaceans. The discovery of this new species adds to the knowledge of the highly endemic benthic fauna of seamounts of the newly created Nazca-Desventuradas Marine Park, emphasizing the relevance of this area for marine conservation.

## Introduction

Although recognized by their high levels of diversity and endemism, the seamounts located in the Salas & Gómez (SGR) and Nazca Ridges (NR), in the Southeastern Pacific (SEP), are among the most remote and least explored marine ecosystems (Parin, Mironov & Nesis, 1997; Poupin, 2003; Poupin, 2008; Gálvez-Larach, 2009; Fernández et al., 2014; Easton et al., 2017). In order to preserve this ecosystem, a marine protected area was created in 2016, the Nazca-Desventuradas Marine Park (NDMP), covering ∼300,000 km^2^ and comprising the intersection of the SGR-NR and the Desventuradas Islands (San Ambrosio and San Felix Islands) within the Chilean exclusive economic zone (EEZ).

The biodiversity information for SGR and NR has been mainly focused outside the Chilean EEZ (mainly west of ∼83°W), where 22 seamounts were explored between 1973 and 1987 by research expeditions from the former Soviet Union (Parin, Mironov & Nesis, 1997), representing only ∼3% of the seamounts that make up both dorsal ranges. Within the Chilean EEZ, the CIMAR 6 cruise (CONA, 2000) and the “Pristine Seas Expedition” (National Geographic OCEANA, 201) multidisciplinary expeditions studied the shallow subtidal zone of Salas & Gómez Island and Desventuradas Islands (DI) and Guyot Stockman (Gálvez-Larach, 2009; Fernández et al., 2014). Each new expedition has added new records and/or new species for science, mainly including crustaceans, echinoderms, fishes and mollusks (Poupin, 2003; Retamal, 2004; Fernández et al., 2014; Easton et al., 2017; Sellanes et al., 2019).

Decapod crustaceans (mainly brachyurans) are among the taxa that included more new species or range extensions through this area in the last years, as reported by Zarenkov (1990; URRS expeditions), Retamal (2001), Retamal (2004) and Retamal & Arana (2016). It remains curious that only a few species of squat lobsters have been ever reported for this vast area, despite the fact that this group is generally abundant and species-rich in several marine ecosystems, particularly in seamounts (Schnabel et al., 2011). *Phylladiorhynchus pusillus* (Henderson, 1885) and *P. integrirostris* (Dana, 1852), belonging to the Galatheidae, have been reported for Easter Island, Salas & Gómez Island and the Juan Fernández archipelago, from the subtidal to 80 m depth (Retamal, 2004; Baba et al., 2008). Some deep-sea species of Munidopsidae, e.g., *Galacantha bellis* Henderson, 1885, *Munidopsis antonii* (Filhol, 1884) have been cited in the Juan Fernández area (Andrade, 1985; Macpherson, 2007), along the East Pacific Rise (Liu, Li & Lin, 2020) and southern French Polynesia seamounts (Macpherson, 2013).

In the present study we describe a new species of *Munida* for the NDMP, collected during the CIMAR 22 expedition. We also provide genetic data of the new species, assessing its phylogenetic relationships with congeners, as well as insight on its habitat, based in underwater imagery obtained with a remotely operated vehicle (ROV).

## Methodology

**Figure 1:**
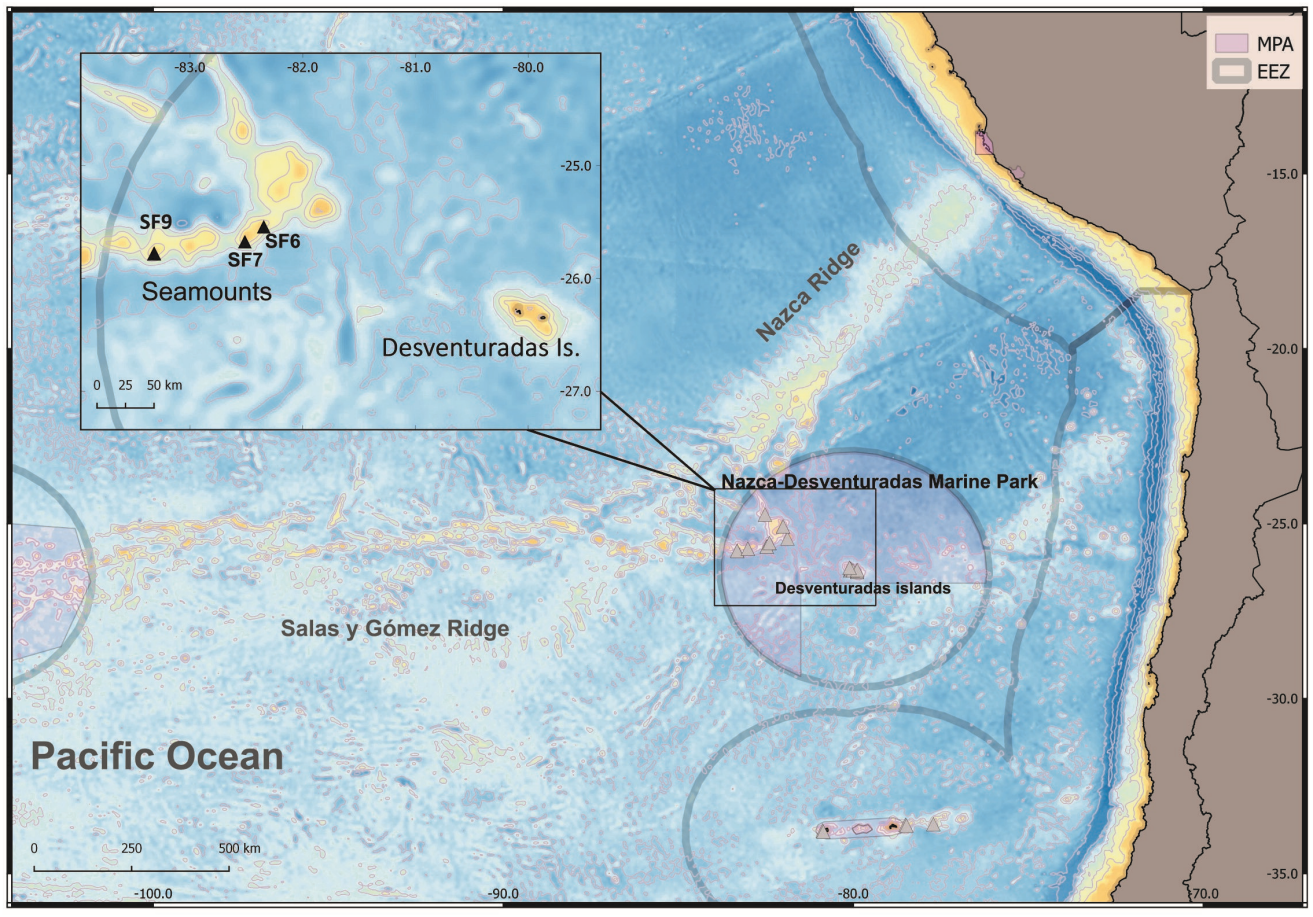
Map of the study area explored during the CIMAR22 cruise, comprising seamounts from Salas & Gómez and Nazca Ridges, Desventuradas Islands and the Juan Fernández Archipelago. Grey lines represent the Chilean exclusive economic zones (EEZ, continental and insular). Grey triangles: sampling points. *Munida diritas* sp. nov. was collected in seamounts SF9 and was observed in situ (ROV, see Video S1) in SF6 and SF7 seamounts. Credits for the map: Ariadna Mecho.

The samples were obtained aboard the research vessel AGS-61 “Cabo de Hornos”, between October 22 and November 11, 2016, during the multidisciplinary oceanographic cruise CIMAR 22 “Oceanic Islands”. The aim of the cruise was to study benthic habitats and fauna of unexplored seamounts of the Juan Fernández and Desventuradas Ecoregion (Fig. 1) (Spalding et al., 2007); ecoregion number 179; Sellanes et al., 2019). Eleven visual observations of the study sites were conducted, using an ROV (Commander MK2; Mariscope Meerestechnik, Kiel, Germany) equipped with a HD Camcorder (Panasonic SD 909) and laser pointers (10 cm apart). Collections were performed at 10 sites (150 to 340 m depth) using a modified Agassiz trawl with a mouth of 1.5 m × 0.5 m (width × height) fitted with a net of 12 mm mesh at the cod end, and operated in 10 min hauls (bottom contact) at ∼3 knots. The collected material was preserved in 95% ethanol (Sellanes et al., 2019). Type material and paratypes specimens were deposited in the MNHNCL, SCBUCN and MNHN. Sample collection was performed under permission Res. Ext N^∘^3685/2016 from SUBPESCA (Chile) to Universidad Católica del Norte.

The terminology employed in the descriptions largely follows Baba et al. (2009) and Macpherson & Baba (2011). The length of the carapace (CL) indicates the postorbital length measured along the dorsal midline from the posterior margin of the orbit to the posterior margin of the carapace. The length of each pereopod article is measured in lateral view along its extensor margin (excluding distal spine), the breadth is measured at its widest portion. Other abbreviations used are: Mxp3 = maxilliped 3; P1, pereopod 1; P2–4, pereopods 2–4.

### Molecular analysis

The protocol described by Macpherson, Rodríguez-Flores & Machordom (2017) was used for DNA extraction: Tissue of one specimen (MNHN-IU-2014-13931) was isolated from the muscle of the fifth pair of pereopods and homogenized overnight with 20 µl proteinase K in 180 µl of buffer ATL (QIAGEN). The extraction was performed using DNeasy Blood and Tissue Kit following manufacturer instructions (QIAGEN). One molecular marker was amplified: a 16S rRNA (16S) fragment, using 16SAR-16SBR from (Palumbi et al., 1991) pair of primers.

The pre-mixing of the PCR reagents was built in 25 µl total volume, which included 2 µl of DNA extracted, 0,2 mM of each deoxyribonucleotide triphosphate (dNTP), 0,2 µM of each primer forward and reverse, 2U of MyTaq polymerase (Bioline), 5 µl of 5x buffer solution with MgCl_2_ and sterilized water. PCR amplification was performed with a thermal cycle including an initial denaturation of 94−95° for 1–4 min and 40 cycles with 95° for 1 min, annealing in 42−45° for 1 min followed by an extension set on 72° for 1 min. A final extension cycle at 72° C was set for 10 min. The amplicons were visualized in agarose 1% gels and purified using ExoSAP-IT™ PCR Product Cleanup Reagent (Thermo Fischer) before sequencing. The purification products were sent to Secugen S.L. (Madrid) for DNA Sanger sequencing (protocol described by Rodríguez-Flores, Macpherson & Machordom, 2020).

The nucleotide sequences (forward and reverse) were visualized and assembled with Sequencher 4.10.1 software package (Gene Codes Corp.). Manual alignment for the 16S genes was carried out in MAFFT (Katoh et al., 2002) and the revised in AliView (Larsson, 2014), (protocol described by (Rodríguez-Flores, Macpherson & Machordom, 2020).

One hundred and fourteen 16S rRNA sequences of *Munida* spp., *Raymunida* spp., *Leiogalathea ascanius, Eumunida sternomaculata*, *Cervimunida johni* and *Pleuroncodes monodon* available in NCBI GenBank (Table S1) were extracted and aligned with the one of *Munida diritas* sp. nov., using default MUSCLE (Edgar, 2004) parameters. In species where there were more than three sequences of this marker, only three were chosen to consider intraspecific genetic variation in the analysis. The resulting alignment of 556 pb was used to construct the maximum likelihood phylogenetic tree with the PHYML 3.0 software (Guindon et al., 2010) and the Geneious Prime 2020 1.1 (Kearse et al., 2012) plugin, using the following settings: bootstrap replicates = 1,000, optimize = Topology/length/rates, Topology search = NNI, nucleotide model substitution= GTR. Significant bootstrap values (>90) are reported at the nodes. *Leiogalathea ascanius*, *Eumunida sternomaculata* and *Raymunida* spp. were used as outgroup. In addition, the locality and biogeographical realm (see Spalding et al., 2007) where each specimen was collected, was included in Table S1.

### Nomenclature

The electronic version of this article in Portable Document Format will represent a published work according to the International Commission on Zoological Nomenclature (ICZN), and hence the new names contained in the electronic version are effectively published under that Code from the electronic edition alone. This published work and the nomenclatural acts it contains have been registered in ZooBank, the online registration system for the ICZN. The ZooBank LSIDs (Life Science Identifiers) can be resolved and the associated information viewed through any standard web browser by appending the LSID to the prefix http://zoobank.org/. The LSID for this publication is: LSID: *Munida diritas* sp. nov. urn:lsid:zoobank.org:pub:4F3C623C-0C27-4AEA-B303-E6A86CF4FEA1. The online version of this work is archived and available from the following digital repositories: PeerJ, PubMed Central and CLOCKSS.

## Results

### Systematic account

**Table:** 

**Figure 2:**
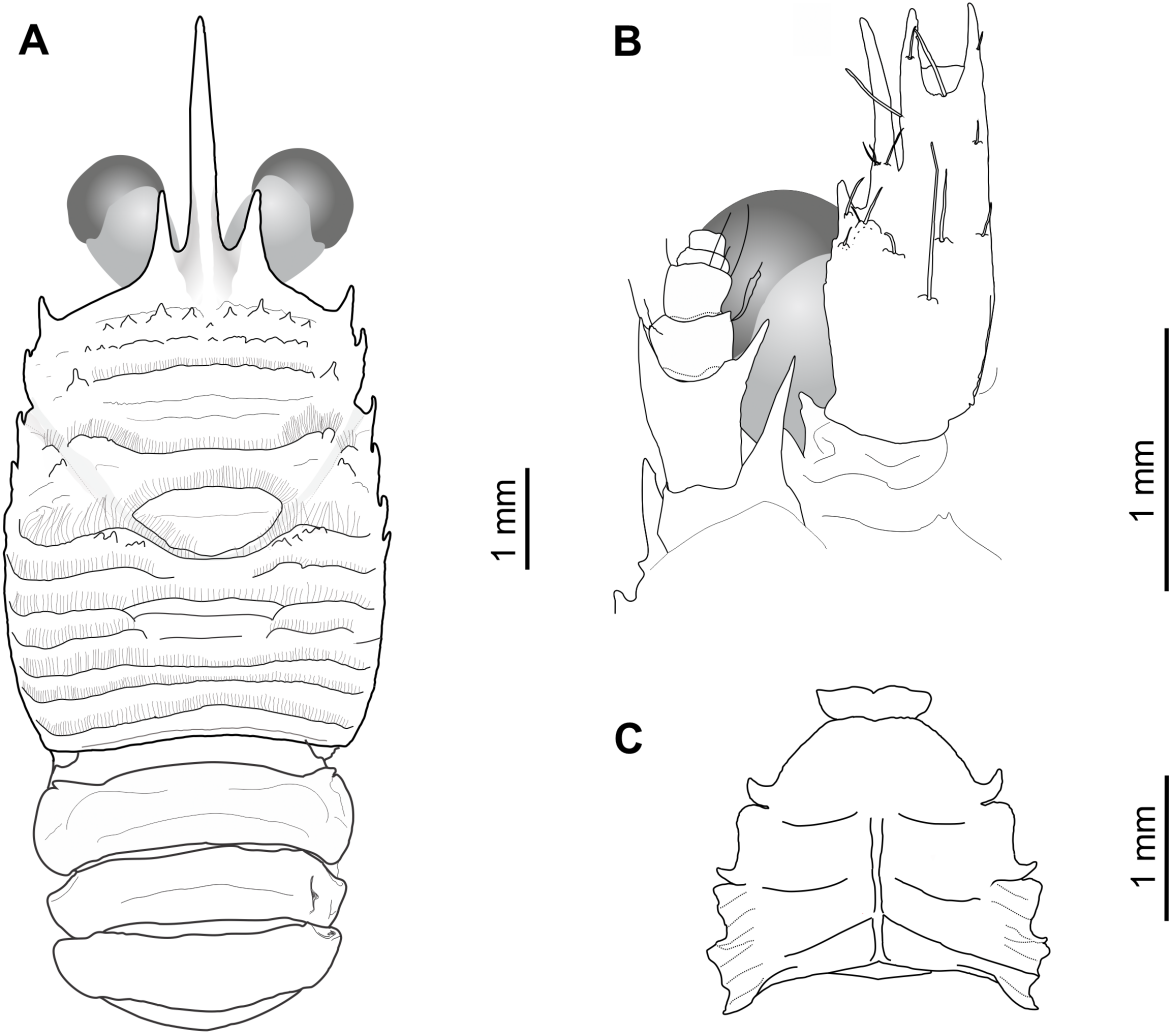
*Munida diritas* sp. nov., holotype, male (CL 4.9 mm), MNHNCL DEC-15175, Seamount SF 9 off Chile; 25°46.8′S, 83°9.6′W; 200 m depth. (A) Carapace, eyes and pleonites 1–4, dorsal view. (B) Left anterolateral part of carapace, eye, first segment of antennular peduncle and antennal peduncle, ventral view. (C) Thoracic sternum, ventral view.

**Figure 3:**
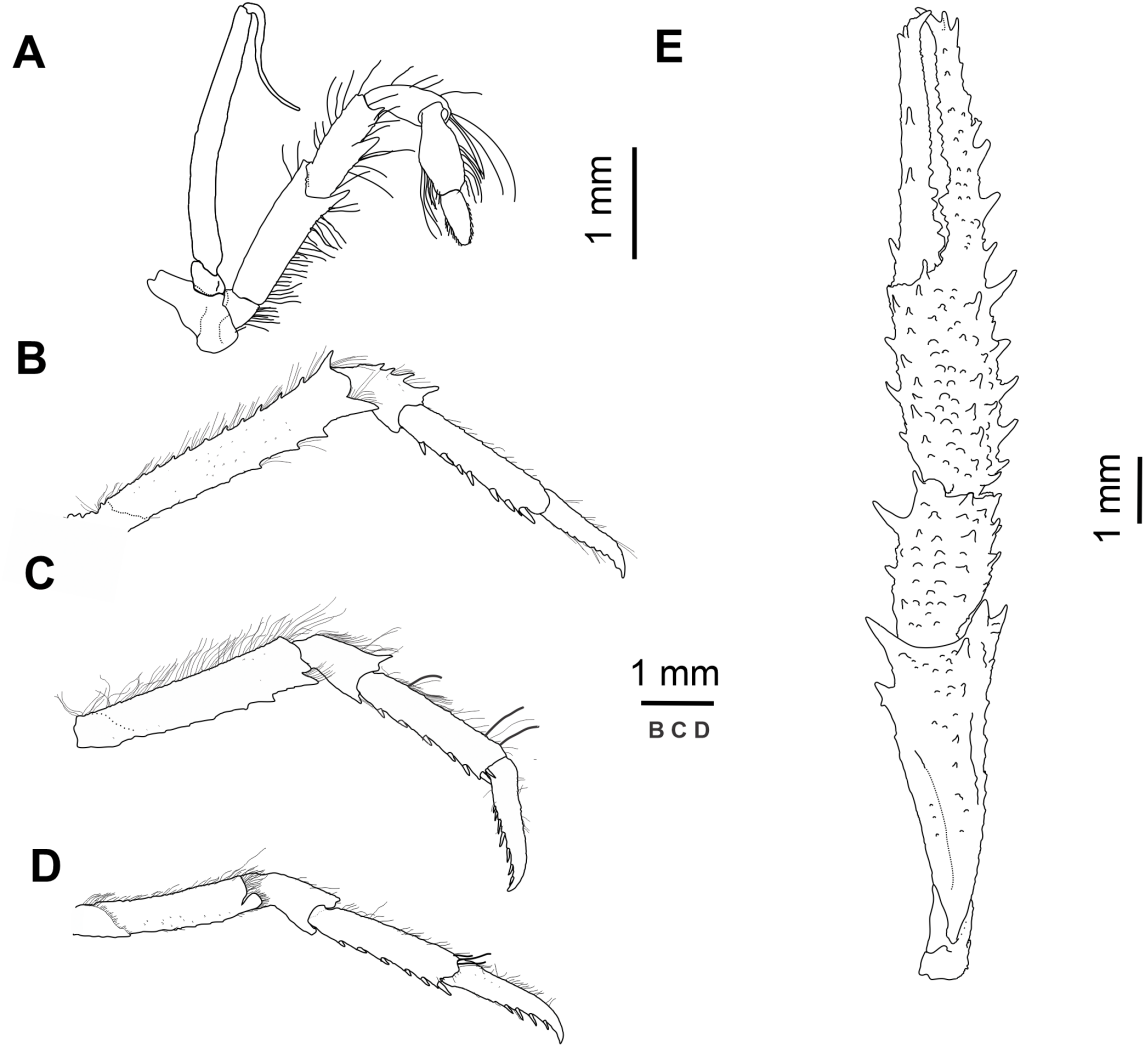
*Munida diritas* sp. nov., holotype, male (CL 4.9 mm), MNHNCL DEC-15175, Seamount SF 9 off Chile; 25°46.8′S, 83°9.6′W; 200 m depth. (A) Right third maxilliped, lateral view. (B) Right second pereopod, lateral view. (C) Right third pereopod, lateral view. (D) Right fourth pereopod, lateral view. (E) Chela and carpus of right cheliped, dorsal view.

Superfamily: Galatheoidea Samouelle, 1819
Family: Munididae Ahyong, Baba, Macpherson & Poore, 2010
Genus: *Munida* Leach, 1820
*Munida diritas* sp. nov. Gallardo & Macpherson
(Figs. 2 and 3)


***Material examined***:

Holotype: MNHNCL DEC-15175 (ex-SCBUCN-7266) (Figs. 2 and 3), CL: 5.0 mm, male, seamount off the coast of Chile, CIMAR 22 cruise, station SF9, 25°46.8′S, 83°9.6′W, 27 October, 2016, ∼200 m depth.

Paratypes: ovigerous female, CL: 4.2 mm (MNHN-IU-2014-13931, ex SCBUCN-8677); male, CL: 3.6 mm (MNHNCL DEC-15176); male, CL: 3.9 mm (SCBUCN-7265); female, CL: 3.5 mm (SCBUCN-7991); ovigerous female, CL: 3.8 mm (SCBUCN-7992); all locations same as holotype.

***Description:*** *Carapace*: Slightly longer than wide. Transverse ridges mostly interrupted without secondary ridges between them. Main transverse ridges on posterior part of carapace interrupted in cardiac region. Ridges with dense short, not iridescent setae, and few scattered long iridescent setae. Gastric region with one row of eight epigastric spines, largest pair just behind supraocular spines. One parahepatic, one postcervical and one branchial dorsal spine on each side. Frontal margins slightly oblique. Lateral margins slightly convex. Anterolateral spine well-developed, situated at anterolateral angle, not reaching to level of sinus between rostrum and supraocular spines; second marginal spine before anterior branch of cervical groove smaller than preceding one. Branchial margins with four spines, decreasing in size posteriorly. Rostrum horizontal, slightly sinuous, about 0.5 times length of remaining carapace. Supraocular spines nearly reaching midlength of rostrum and clearly not reaching distal corneal margins, subparallel and slightly directed upwards.

*Sternum*: Sternite 4 trapezoidal, with few short striae, anterior margin widely contiguous to sternite 3. Distinct short carinae on lateral surfaces of sternites 6 and 7.

*Pleon*: Pleonites 2 and 3 each with one transverse ridge behind anterior ridge; anterior ridge of somite 2 unarmed; pleonite 4 and 5 smooth; posteromedian margin of pleonite 6 straight.

*Eyes*: Small, maximum corneal diameter 0.4 distance between bases of anterolateral spines.

*Antennule*: Article 1 with 2 well-developed subequal distal spines; two spines on lateralmargin, proximal one short, located at midlength of article, distal much longer than proximal and reaching end of distal spines.

*Antenna*: Article 1 with 1 strong distal spine on mesial margin, reaching distal margin of article 2. Article 2 with 2 long distal spines on mesial and lateral margins, nearly reaching end of article 3. Penultimate article unarmed.

*Mxp3*: Ischium with small distal spine on flexor margin. Merus shorter than ischium, measured along extensor margin; bearing 2 well developed spines on flexor margin, proximal spine stronger than distal; extensor margin unarmed. Carpus unarmed.

*P1*: Squamous, with numerous long iridescent and non-plumose setae, more dense on mesial, lateral and dorsal borders of articles. P1 twice carapace length, merus 0.8 length of carapace, twice as long as carpus, with some strong distal spines, distomesial spine not reaching proximal third of carpus. Carpus 0.9 length of palm, 1.5 times as long as broad, with several spines scattered along mesial and dorsal sides. Palm 1.5 times longer than broad, with row of dorsal spines; lateral margin with row of spines extending onto fixed finger;; mesial margin with row of spines., continuing along mesial margin of movable finger. Fingers slightly longer than palm, dactylus with proximal and subdistal spines on mesial margin and two widely separated spines on dorsal surface adjacent to mesial margin.

*P2–4*: Moderately long and slender, furnished with long plumose and iridiscent setae along extensor margin of articles. P2 1.3 times carapace length. Meri shorter posteriorly (P3 merus 0.8 length of P2 merus, P4 merus 0.7 length of P3 merus); P2 merus 0.9 length of carapace, 4.5–5.0 times as long as high, 1.2–1.5 times longer than P2 propodus; P3 merus 4.0–5.0 times longer than high, 1.2–1.4 times longer than P3 propodus; P4 merus 4.0 times as long as high, as long as P4 propodus. Extensor margins of P2-3 meri with row of proximally diminishing spines, and unarmed on P4, distal spine prominrnt; flexor margins distally with one prominent spine followed proximally by several eminences; lateral sides unarmed. Carpi with 2-4 spines on extensor margin of P2-3, unarmed on P4; flexor margin with distal spine. Propodi 4.0–4.5 (P2-3)-4.0 (P4) times as long as high; extensor margin unarmed; 6-7 slender movable spines along flexor margin of P2-4. Dactyli slender, extensor border slightly convex on proximal half, slightly curving distally, length 0.8–0.9 that of propodi; flexor margin with 7–8 movable spinules along entire border; P2 dactylus 5 times longer than wide.

***Coloration***: In fresh condition, body entirely white (see additional material Video S1).

***Genetic data*:** 16S GENBANK CODE GenBank (accession number: MT936349).

***Remarks:*** The new species belongs to the group of species with 3–4 branchial spines on the carapace and lateral carinae on the 6–7 thoracic sternites. This group includes only species from the Indian Ocean and Western-Central Pacific waters: *M. cristulata* Macpherson, Rodríguez-Flores & Machordom, 2017, *M. ignea* Macpherson, 2006, M. lenticularis Macpherson & De Saint Laurent, 1991 *M. maculata* Komai, 2012, *M. muscae* Macpherson & De Saint Laurent, 2002, *M. ommata* Macpherson, 2004, *M. psylla* Macpherson, 1996, *M. rufiantennulata* Baba, 1969 and *M. vicina* Komai, 2012. From this group of species the closest relatives are: *M. muscae*, from Madagascar, Reunion Island and Mozambique Channel, *M. psylla*, from Papua-New Guinea, New Caledonia and New Zealand, and *M. vicina*, from Kurose Bank and Izu Islands (Japan), characterized by an unarmed pleon.

*Munida psylla* is distinct from the new species in having the distomesial spine of the antennal article 2 overreaching the end of the article 4, whereas this spine at most ends at the end of article 3 in the new species. Furthermore, the P1 fixed finger is lacking spines along the lateral margin, other than subterminal spines, in *M. psylla*, whereas there is a row of spines along the lateral margin of the P1 fixed finger in *M. diritas*.

The new species is closely related to *Munida vicina*, but both are distinguished by several characters (see the description and illustration by Komai (2012):

• The distomesial spine of the antennal article 2 barely reaches the end of the article 3 in the new species, whereas this spine clearly exceeds this article in *M. vicina*.
• The P1 (chelipeds) are more spinose, having also stronger spines, in the new species than in *M. vicina*. The P1 movable and fixed fingers have several spines along the entire mesial and lateral margins, respectively, in the new species, whereas the fingers only have proximal and distal spines in *M. vicina*. Furthermore, the spines along the lateral margin of the palm and fixed finger are clearly larger in the new species than in *M. vicina*.

*Munida diritas* is distinct from *M. muscae* by the following aspects:

• The distomesial spine of the antennular article 1 is shorter than the distolateral in *M. muscae*, whereas they are subequal in *M. diritas*.
• The movable finger of the P1 (chelipeds) has a row of spines along the mesial margin in *M. diritas*, whereas this finger only has proximal and distal spines in *M. muscae.* Furthermore, as it was observed in *M. vicina*, the spines along the lateral margin of the palm and fixed finger are clearly larger in the new species than in *M. muscae*.

**Figure 4:**
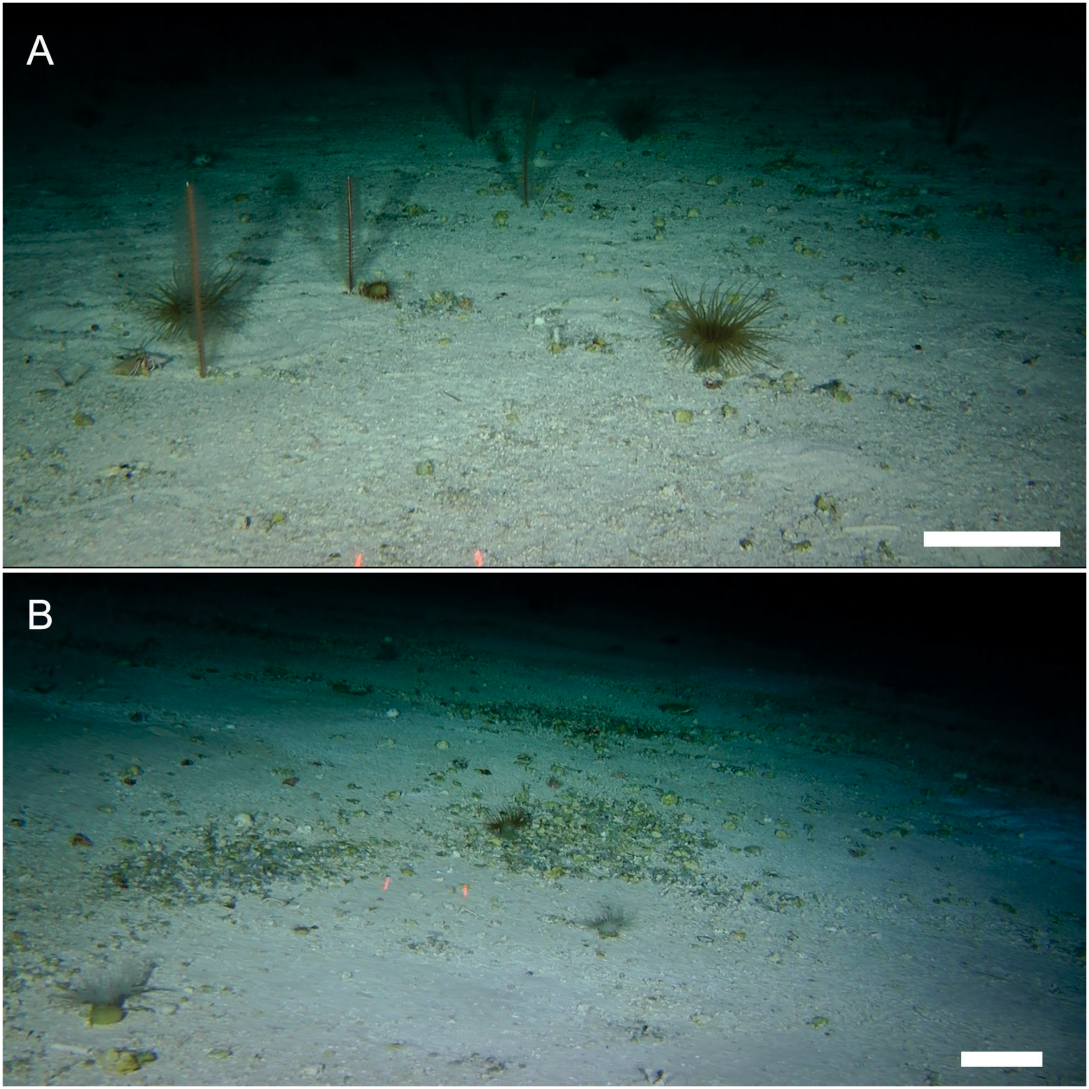
Benthic microhabitats of *Munida diritas* sp. nov. Images taken with an ROV at the sites where *M. diritas* sp. nov. was spotted within the Nazca-Desventuradas Marine Park. (A) Seamount SF7, 176 m depth, regular continuous homogeneous bottom with little relief, coarse sand and some rhodoliths characterized by sea pens (*Protoptilum* sp.), anemones (*Hormathia* sp. and Cerianthids). (B) Seamount SF9, 200 m depth, regular continuous homogeneous bottom with coarse sand and rhodoliths, dominated by sponges and anemones (*Hormathia* sp. and Cerianthids). Scale bar: 10 cm. Image credits for A and B: Matthias Gorny-OCEANA.

***Distribution, habitat and ecological aspects***: Apart from the type locality, as suggested by ROV observations, the new species could be present at two other seamounts in the intersection of NR and SGR: Stations SF6 (25°33′S, 82°23′^∘^W) and SF7 (25°39′S, 82°28′W) (Fig. 4), 180 and 176 m depth, respectively. We base our conjecture on the general morphology of the cephalothorax and P1, to identify the new species in the ROV images. The bottom at SF7 and SF9 seamounts was relatively homogeneus, with little relief and dominated by mixed sediment (coarse sand, maërl-rhodoliths: unattached nodules of crustose coralline red algae, sponges, and pteropod shell-beds) (Fig. 4). In ROV observations, *M. diritas* sp. nov. was found associated to a microhabitat of anemones (*Hormathia* sp. and Ceriantharia), hydrozoan colonies, polychaete tubes (*Lanice* sp.) and sea urchin tests (e.g., *Stereocidaris nascaensis*) (Fig. 4). The new species was observed hunting mysidaceans that foraged around anemones’ tentacles. It seems that the white coloring of the new species favors camouflage with sediment, using microtopographic features and resources of microhabitat to block visual recognition of preys in ambush tactics (Video S1).

***Etymology*:** From the Latin “*diritas”* (=unfortunate, misfortune), alluding to the type locality at seamounts near Desventuradas Islands (Nazca-Desventuradas Marine Park). “Desventurado” in Spanish means: suffering from misfortune.

**Figure 5:**
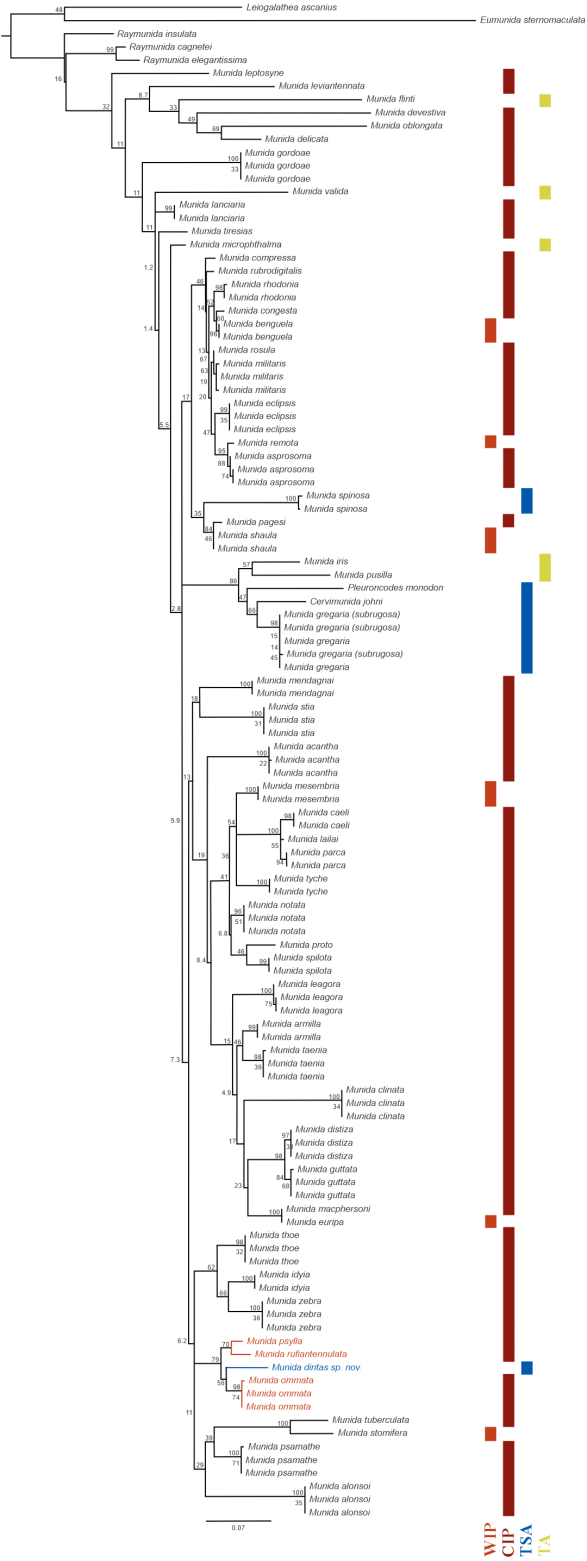
Phylogenetic relationships of *Munida diritas* sp. nov. with other *Munida* spp. based on 16S rRNA. The reconstructed ML phylogram and node bootstrap values are shown. *Leiogalathea ascanius, Eumunida sternomaculata*, and *Raymunida* spp. were used as outgroup taxa. Scale bar indicates the number of substitutions/site. The colored bars indicate the origin of the sequenced samples. Biogeographic realms (Spalding et al., 2007): WIP = West Indo-Pacific Ocean, CIP = Central Indo-Pacific Ocean, TA = Tropical Atlantic, TSA = Temperate South America.

***Phylogenetic relationships:*** The 16S rRNA sequence of *Munida diritas* sp. nov. is more similar to *M. ommata* (94.6–94.8%), *M. rufiantennulata* (94.7%) and *M. psylla* (95.3%) than to species in the Southeast Pacific, such *M. gregaria/subrugosa* (89.7–90.0%), *Cervimunida johni* (88.5%) and *Pleuroncodes monodon* (86.4%). Furthermore, the *Munida diritas* sp. nov. sequence appears to form a clade with Central Indian Pacific species (*Munida ommata*, *Munida rufiantennulata* and *Munida psylla,* Fig. 5) while species inhabiting the Southeast Pacific (TSA, Temperate South America) group in another clade, despite belonging to other genera (Fig. 5). The sequences of *Cervimunida johni*, *Pleuroncodes monodon*, and *M. gregaria/subrugosa* differed by 10 to13.6% from the sequence of *Munida diritas* sp. nov.

## Discussion

The genetic analysis using 16S rRNA sequences suggests that *Munida diritas* sp. nov. is close to *M. ommata, M. rufiantennulata* and *M. pusylla*, all are characterized by having lateral carinae on the thoracic sternum. The genetic distances among those four species range from 4.7 to 5.2%. It should be noted that, among the group characterized by the presence of thoracic sternal carinae, these are the three species for which sequence data is available.

The genetic distances observed between the new species and other species of *Munida* occurring along the west coast of America, i.e., *M. gregaria, M. quadrispina*, *Pleuroncodes monodon*, were larger than 10% for 16S (Fig. 5). These values imply high levels of genetic divergence, even exceeding the mean divergence reported for other squat lobsters (Machordom & Macpherson, 2004; Rodríguez-Flores et al., 2019), and indicating a different phylogenetic origin. The obtained tree supports the existence of a very old common ancestor, with closer relationships with Indo-Pacific species than with American species (Fig. 5).

*Munida diritas* is the fifth species of the genus *Munida* reported for Chilean waters (Bahamonde & López, 1962; Hendrickx, 2003; Baba et al., 2008), and the first for subtropical waters in seamounts of the Juan Fernández and Desventuradas Ecoregion. Other species of *Munida* that occur along the continental shelf and slope off Chile (Hendrickx, 2003; Baba et al., 2008) are: *M. curvipes* Benedict 1902, *M. gregaria* (Fabricius 1793), *M. montemaris* Bahamonde & López, 1962, and *M. propinqua* Faxon 1893. The new species remarkably differs in morphology from them, while is closer to some species occurring in the Indo West Pacific and French Polynesia. Phylogenetic analysis using 16S clusters the new species with *M. ommata, M. psylla* and *M. rufiantennulata,* all characterized by the possession of lateral carinae on the thoracic sternites 6–7. Indeed, the new species is similar also to other species characterized by the possession of four or less branchial spines and the lateral carinae at least on the thoracic sternites 6–7 (Macpherson, 1994; Machordom & Macpherson, 2004; Baba et al., 2009; Komai, 2012), this last character probably related to reproductive behaviour (Machordom & Macpherson, 2004). Other Munididae species described for French Polynesia, e.g., *Babamunida plexaura* Macpherson & De Saint Laurent, 1991, *Munida rubella* Macpherson & De Saint Laurent, 1991, *M. rubrovata* Macpherson & De Saint Laurent, 1991, have been recently collected around the Salas & Gómez Ridge seamounts (M.A. Gallardo, unpublished data). In addition, up to 40% of the crustaceans of this area (e.g., decapods and stomatopods listed by Poupin (2008) are Indo-West Pacific species (subtropical origin). All this evidence indicates that this area is biogeographically very different from the continental margin of the SE Pacific, probably due to the change of environmental conditions observed at ∼80−85°W. In this area the influence of the cold and productive Humboldt Current System vanishes and the intrusion of subtropical oligotrophic waters from the West begins (Fuenzalida et al., 2007; Thiel et al., 2007). Factors such as temperature, salinity, oxygen and food influence the biogeographic distribution of marine ectotherms (Pörtner, 2002; Pörtner, 2010), it is thus expected that species of subtropical origin would limit their distribution in cold waters characteristic of the SE Pacific.

The habitat of *Munida diritas* sp. nov. is shared with anemones, hydrozoan colonies, and other filter-feeding organisms commonly inhabiting the seamounts (Easton et al., 2019; Tapia JM, 2020, unpublished data), forming microhabitat for different taxa (Tapia JM, 2020, unpublished data). These microhabitat can play an important role in the distribution and abundance of the species they host (Buhl-Mortensen & Mortensen, 2004; Cordes et al., 2008), influencing also its feeding behaviour (Becker et al., 2009). The in situ video images show that *Munida diritas* sp. nov. could be an active predator, as it has been observed in other species of *Munida*, e.g., *M. sarsi* (Hudson & Wigham, 2003; Lovrich & Thiel, 2011). These relationships (between microhabitat and associated species) emphasise the role of these seamounts in the maintenance of biodiversity, and the importance of the conservation of this unique biodiversity hotspot.

## Conclusion

We describe *Munida diritas* sp. nov. from seamounts of Nazca-Desventuradas Marine Park, based on morphological and phylogenetic studies. Molecular and morphological data indicates that the new species remarkably differs from other species from the continental margin of the SE Pacific, and it is closer to some species occurring in the Indo Pacific and French Polynesia.

##  Supplemental Information

10.7717/peerj.10531/supp-1 Supplemental Information 1 16S rRNA sequences used for the phylogenetic analysis Species, Genbank accession numbers, length, biogeographical reals (see Spalding et al., 2007), Locality (origin of each specimen) and references.

10.7717/peerj.10531/supp-2 Supplemental Information 2 16S rRNA sequences. of *Munida diritas sp nov.*

10.7717/peerj.10531/supp-3 Supplemental Information 3 Benthic microhabitats of *Munida diritas* sp. nov. Video taken with an ROV at the seamount SF7 where *M. diritas* sp. nov. was spotted within the Nazca-Desventuradas Marine Park Video Credits: Matthias Gorny OCEANA

## Abbreviations

MNHN: Muséum National d‘Histoire Naturelle, Paris, France
MNHNCL: Museo Nacional de Historia Natural, Chile
NDMP: Nazca Desventuradas Marine Park
ROV: Remotely operated underwater vehicle
SCBUCN: Sala de Colecciones Biológicas de la Universidad Católica del Norte, Chile

## Additional Information and Declarations

### Field Study Permissions

The following information was supplied relating to field study approvals (i.e., approving body and any reference numbers): Sample collection was performed under permission Res. Ext N° 3685/2016 from SUBPESCA (Chile) to Universidad Católica del Norte.

### DNA Deposition

The following information was supplied regarding the deposition of DNA sequences: The 16S sequence are available in the Supplemental Files and at GenBank: MT936349 (Munida sp. 1 MDLAGS-2020)
https://www.ncbi.nlm.nih.gov/nuccore/1896876245.
https://www.ncbi.nlm.nih.gov/Taxonomy/Browser/wwwtax.cgi?mode=Info&id=2767541&lvl=3&lin=f&keep=1&srchmode=1&unlock.

### Data Availability

The following information was supplied regarding data availability: The specimens for study are deposited in the following collections: - Museo Nacional de Historia Natural (MNHNCL), Chile. Holotype MNHNCL DEC-15175 , Paratype 2 MNHNCL DEC-15176. - Muséum National d‘Histoire Naturelle, Paris, France. Paratype 1 MNHN-IU-2014-13931. - Sala de Colecciones Biológicas UCN, Coquimbo, Chile. Paratype 3 SCBUCN-7265, Paratype 4 SCBUCN-7991, Paratype 5 SCBUCN-7992.

### New Species Registration

The following information was supplied regarding the registration of a newly described species: Publication LSID: urn:lsid:zoobank.org:pub:4F3C623C-0C27-4AEA-B303-E6A86CF4FEA1 *Munida diritas* sp. nov.: urn:lsid:zoobank.org:act:0EC498DE-46D1-42B8-95D3-E6CDD4FBA20F.
