# Supplementary material for: A new species of Munida Leach, 1820 (Crustacea: Decapoda: Anomura: Munididae) from seamounts of the Nazca-Desventuradas Marine Park
Source: PeerJ. 2021 Jan 5;9:e10531. doi: 10.7717/peerj.10531 (PMC7792524; doi:10.7717/peerj.10531)
Supplement: Supplemental Information 1 — Species, Genbank accession numbers, length, biogeographical reals (see Spalding et al., 2007), Locality (origin of each specimen) and references. [file peerj-09-10531-s001.pdf]

Supplementary Material

Table S1. 16S rRNA sequences used for the phylogenetic analysis. Species, Genbank accession numbers, length, biogeographical reals (see Spalding 2007), Locality (origin of each specimen) and references.

Species	Genebank accession number	Length (pb)	*Biogeographic realms*	Locality	Reference
Munida acantha	AY351095	513	Central Indo-Pacific	New Caledonia	Machordom & Macpherson 2004
Munida acantha	AY351096	513	Central Indo-Pacific	New Caledonia	Machordom & Macpherson 2004
Munida acantha	AY351097	513	Central Indo-Pacific	New Caledonia	Machordom & Macpherson 2004
Munida alonsoi	AY351098	513	Central Indo-Pacific	New Caledonia	Machordom & Macpherson 2004
Munida alonsoi	AY351102	513	Central Indo-Pacific	New Caledonia	Machordom & Macpherson 2004
Munida alonsoi	AY351104	513	Central Indo-Pacific	New Caledonia	Machordom & Macpherson 2004
Munida armilla	AY351106	513	Central Indo-Pacific	New Caledonia	Machordom & Macpherson 2004
Munida armilla	AY351107	514	Central Indo-Pacific	New Caledonia	Machordom & Macpherson 2004
Munida asprosoma	JF727277	446	Central Indo-Pacific	Taiwan	Cabezas et al. 2011
Munida asprosoma	JF727279	446	Central Indo-Pacific	Taiwan	Cabezas et al. 2011
Munida asprosoma	JF727280	446	Central Indo-Pacific	Taiwan	Cabezas et al. 2011
Munida caeli	EU417975	430	Central Indo-Pacific	Solomon Islands	Cabezas et al. 2009
Munida caeli	EU417977	430	Central Indo-Pacific	Solomon Islands	Cabezas et al. 2009
Munida clinata	AY351111	516	Central Indo-Pacific	New Caledonia	Machordom & Macpherson 2004
Munida clinata	AY351112	516	Central Indo-Pacific	New Caledonia	Machordom & Macpherson 2004
Munida clinata	AY351113	516	Central Indo-Pacific	New Caledonia	Machordom & Macpherson 2004
Munida compressa	AY351114	513	Central Indo-Pacific	Salomon Is.	Machordom & Macpherson 2004
Munida congesta	AY351115	512	Central Indo-Pacific	New Caledonia	Machordom & Macpherson 2004
Munida delicata	EU417976	434	Central Indo-Pacific	New Caledonia	Cabezas et al. 2009
Munida devestiva	EU417989	427	Central Indo-Pacific	New Caledonia	Cabezas et al. 2009
Munida distiza	AY351116	512	Central Indo-Pacific	New Caledonia	Machordom & Macpherson 2004
Munida distiza	AY351118	512	Central Indo-Pacific	New Caledonia	Machordom & Macpherson 2004
Munida distiza	AY351119	512	Central Indo-Pacific	New Caledonia	Machordom & Macpherson 2004
Munida eclepsis	AY351120	512	Central Indo-Pacific	New Caledonia	Machordom & Macpherson 2004
Munida eclepsis	AY351121	512	Central Indo-Pacific	New Caledonia	Machordom & Macpherson 2004
Munida eclepsis	AY351122	512	Central Indo-Pacific	New Caledonia	Machordom & Macpherson 2004
Munida gordoae	AY351124	515	Central Indo-Pacific	New Caledonia	Machordom & Macpherson 2004
Munida gordoae	AY351126	515	Central Indo-Pacific	New Caledonia	Machordom & Macpherson 2004

Munida gordoae	AY351127	515	Central Indo-Pacific	New Caledonia	Machordon & Macpherson 2004
Munida guttata	AY351128	512	Central Indo-Pacific	New Caledonia	Machordon & Macpherson 2004
Munida guttata	AY351130	512	Central Indo-Pacific	New Caledonia	Machordon & Macpherson 2004
Munida guttata	AY351131	512	Central Indo-Pacific	New Caledonia	Machordon & Macpherson 2004
Munida idyia	MK458565	512	Central Indo-Pacific	Papua New Guinea	(Macpherson et al. 2020)
Munida idyia	MK458566	512	Central Indo-Pacific	Papua New Guinea	Macpherson et al 2020
Munida lailai	EU417988	432	Central Indo-Pacific	Fiji Islands	Cabezas et al. 2009
Munida lanciaria	JF727288	447	Central Indo-Pacific	Taiwan	Cabezas et al. 2011
Munida lanciaria	JF727289	447	Central Indo-Pacific	Taiwan	Cabezas et al. 2011
Munida leagora	AY351132	513	Central Indo-Pacific	New Caledonia	Machordon & Macpherson 2004
Munida leagora	AY351133	513	Central Indo-Pacific	New Caledonia	Machordon & Macpherson 2004
Munida leagora	AY351135	513	Central Indo-Pacific	New Caledonia	Machordon & Macpherson 2004
Munida leptosyne	AY351141	511	Central Indo-Pacific	New Caledonia	Machordon & Macpherson 2004
Munida leviantennata	AY351142	513	Central Indo-Pacific	New Caledonia	Machordon & Macpherson 2004
Munida macphersoni	JF727284	447	Central Indo-Pacific	Taiwan	Cabezas et al. 2011
Munida mendagnai	EU417973	428	Central Indo-Pacific	Solomon Islands	Cabezas et al. 2009
Munida mendagnai	EU417974	428	Central Indo-Pacific	Solomon Islands	Cabezas et al. 2009
Munida militaris	AY351143	512	Central Indo-Pacific	New Caledonia	Machordon & Macpherson 2004
Munida militaris	JF727285	446	Central Indo-Pacific	Taiwan	Cabezas et al. 2011
Munida militaris	JF727286	446	Central Indo-Pacific	Taiwan	Cabezas et al. 2011
Munida notata	AY351144	515	Central Indo-Pacific	New Caledonia	Machordon & Macpherson 2004
Munida notata	AY351146	515	Central Indo-Pacific	New Caledonia	Machordon & Macpherson 2004
Munida notata	AY351147	507	Central Indo-Pacific	New Caledonia	Machordon & Macpherson 2004
Munida oblongata	EU417978	434	Central Indo-Pacific	Solomon Islands	Cabezas et al. 2009
Munida ommata	AY351148	514	Central Indo-Pacific	New Caledonia	Machordon & Macpherson 2004
Munida ommata	AY351150	514	Central Indo-Pacific	New Caledonia	Machordon & Macpherson 2004
Munida ommata	AY351151	514	Central Indo-Pacific	New Caledonia	Machordon & Macpherson 2004
Munida pagesi	AY351152	437	Central Indo-Pacific	New Caledonia	Machordon & Macpherson 2004
Munida parca	EU417986	429	Central Indo-Pacific	New Caledonia	Cabezas et al. 2009
Munida parca	EU417987	431	Central Indo-Pacific	New Caledonia	Cabezas et al. 2009
Munida proto	AY351153	444	Central Indo-Pacific	Fiji Is	Machordon & Macpherson 2004
Munida psamathe	AY351154	511	Central Indo-Pacific	New Caledonia	Machordon & Macpherson 2004
Munida psamathe	AY351155	511	Central Indo-Pacific	New Caledonia	Machordon & Macpherson 2004

Munida psamathe	AY351158	511	Central Indo-Pacific	New Caledonia	Machordon & Macpherson 2004
Munida psylla	AY351159	511	Central Indo-Pacific	New Caledonia	Machordon & Macpherson 2004
Munida rhodonia	AY351160	512	Central Indo-Pacific	New Caledonia	Machordon & Macpherson 2004
Munida rhodonia	AY351161	512	Central Indo-Pacific	New Caledonia	Machordon & Macpherson 2004
Munida rosula	AY351162	512	Central Indo-Pacific	New Caledonia	Machordon & Macpherson 2004
Munida rubrodigitalis	AY351163	513	Central Indo-Pacific	New Caledonia	Machordon & Macpherson 2004
Munida rufiantennulata	AY351164	512	Central Indo-Pacific	New Caledonia	Machordon & Macpherson 2004
Munida spilota	AY351165	512	Central Indo-Pacific	New Caledonia	Machordon & Macpherson 2004
Munida spilota	AY351166	512	Central Indo-Pacific	New Caledonia	Machordon & Macpherson 2004
Munida stia	AY351168	517	Central Indo-Pacific	New Caledonia	Machordon & Macpherson 2004
Munida stia	AY351169	517	Central Indo-Pacific	New Caledonia	Machordon & Macpherson 2004
Munida stia	AY351170	517	Central Indo-Pacific	New Caledonia	Machordon & Macpherson 2004
Munida taenia	AY351173	512	Central Indo-Pacific	New Caledonia	Machordon & Macpherson 2004
Munida taenia	AY351175	512	Central Indo-Pacific	New Caledonia	Machordon & Macpherson 2004
Munida taenia	AY351176	512	Central Indo-Pacific	New Caledonia	Machordon & Macpherson 2004
Munida thoe	AY351178	512	Central Indo-Pacific	New Caledonia	Machordon & Macpherson 2004
Munida thoe	AY351179	512	Central Indo-Pacific	New Caledonia	Machordon & Macpherson 2004
Munida thoe	AY351180	512	Central Indo-Pacific	New Caledonia	Machordon & Macpherson 2004
Munida tiresias	AY351183	513	Central Indo-Pacific	New Caledonia	Machordon & Macpherson 2004
Munida tuberculata	AY351184	514	Central Indo-Pacific	New Caledonia	Machordon & Macpherson 2004
Munida tyche	AY351185	513	Central Indo-Pacific	Vanuatu	Machordon & Macpherson 2004
Munida tyche	AY351186	512	Central Indo-Pacific	New Caledonia	Machordon & Macpherson 2004
Munida zebra	AY351187	511	Central Indo-Pacific	New Caledonia	Machordon & Macpherson 2004
Munida zebra	AY351189	511	Central Indo-Pacific	New Caledonia	Machordon & Macpherson 2004
Munida zebra	AY351190	511	Central Indo-Pacific	New Caledonia	Machordon & Macpherson 2004
Munida diritas sp. nov.	**MT936349**	512	Temperate South America	Desventuras Island	Present study
Munida gregaria	AY700158	416	Temperate South America	Chile	Pérez-Barros et al. 2008
Munida gregaria	EF428963	512	Temperate South America	Argentina	Terrat et al. 2008
Munida spinosa	KJ957175	468	Temperate South America	Argentina	GenBank
Munida spinosa	KJ957182	462	Temperate South America	Argentina	GenBank
Munida subrugosa	AY050075	407	Temperate South America	Chile	Pérez-losada et al. 2002
Munida subrugosa	AY700159	416	Temperate South America	Chile	Pérez-Barros et al. 2008
Munida subrugosa	AY700161	416	Temperate South America	Chile	Pérez-Barros et al. 2008

Munida flinti	MF490158	517	Tropical Atlantic	Brasil	Mantelatto et al. 2017
Munida iris	KF182521	448	Tropical Atlantic	Musean specimen	Bracken-Grissom et al. 2013
Munida microphthalma	MF490159	512	Tropical Atlantic	Brasil	Mantelatto et al 2017
Munida pusilla	KF182522	517	Tropical Atlantic	Musean specimen	Bracken-Grissom et al 2013
Munida valida	JN800548	480	Tropical Atlantic	Musean specimen	Bybee et al. 2011
Munida benguela	KY230467	497	Western Indo-Pacific	Mozambique	Macpherson et al. 2017
Munida benguela	KY230468	501	Western Indo-Pacific	Mozambique	Macpherson et al. 2017
Munida euripa	KY230469	513	Western Indo-Pacific	Madagascar	Macpherson et al. 2017
Munida mesembria	KY230470	513	Western Indo-Pacific	Mozambique	Macpherson et al. 2017
Munida mesembria	KY230471	481	Western Indo-Pacific	Mozambique	Macpherson et al. 2017
Munida remota	KY230472	512	Western Indo-Pacific	Madagascar	Macpherson et al. 2017
Munida shaula	KY230473	512	Western Indo-Pacific	Madagascar	Macpherson et al. 2017
Munida shaula	KY230474	512	Western Indo-Pacific	Madagascar	Macpherson et al. 2017
Munida stomifera	KY230475	477	Western Indo-Pacific	Madagascar	Macpherson et al. 2017
Cervimunida johni	AY351244	513	Temperate South America	Chile	Machordon & Macpherson 2004
Pleuroncodes monodon	AY351259	514	Temperate South America	Chile	Machordon & Macpherson 2004
Raymunida insulata	AY351235	513	Western Indo-Pacific	Seychelles Is	Machordon & Macpherson 2004
Raymunida elegantissima	AY351231	512	Central Indo-Pacific	New Caledonia	Machordon & Macpherson 2004
Raymunida cagnetei	AY351224	511	Eastern Indo-Pacific	Marquesas Is.	Machordon & Macpherson 2004
Eumunida sternomaculata	AY351260	515	Central Indo-Pacific	New Caledonia	Machordon & Macpherson 2004
Leiogalathea laevirostris	AY351252	506	Central Indo-Pacific	New Caledonia	Machordon & Macpherson 2004

References:

Bracken-Grissom, H. D., M. E. Cannon, P. Cabezas, R. M. Feldmann, C. E. Schweitzer, S. T. Ahyong, D. L. Felder, R. Lemaitre, and K. A. Crandall. 2013. A comprehensive and integrative reconstruction of evolutionary history for Anomura (Crustacea: Decapoda). BMC Evolutionary Biology 13:128.

Bybee, S. M., H. Bracken-Grissom, B. D. Haynes, R. A. Hermansen, R. L. Byers, M. J. Clement, J. A. Udall, E. R. Wilcox, and K. A. Crandall. 2011. Targeted Amplicon Sequencing (TAS): A Scalable Next-Gen Approach to Multilocus, Multitaxa Phylogenetics. Genome Biology and Evolution 3:1312–1323.

Cabezas, P., C. Lin, and T. Chan. 2011. Two new species of the deep-sea squat lobster genus

Munida Leach, 1820 (Crustacea: Decapoda: Munididae) from Taiwan: morphological and molecular evidence. Zootaxa 3036:26–38.

Cabezas, P., E. Macpherson, and A. Machordom. 2009. Morphological and molecular description of new species of squat lobster (Crustacea: Decapoda: Galatheidae) from the Solomon and Fiji Islands (South-West Pacific). Zoological Journal of the Linnean Society 156:465–493.

Machordom, A., and E. Macpherson. 2004. Rapid radiation and cryptic speciation in squat lobsters of the genus *Munida* (Crustacea, Decapoda) and related genera in the South West Pacific: molecular and morphological evidence. Molecular Biology and Evolution 33:259–279.

Macpherson, E., P. C. Rodríguez-Flores, and A. Machordom. 2017. New sibling species and new occurrences of squat lobsters (Crustacea, Decapoda) from the western Indian Ocean. European Journal of Taxonomy 343:1–61.

Macpherson, E., P. C. Rodríguez-Flores, and A. Machordom. 2020. Squat lobsters of the families Munididae and Munidopsidae from Papua New Guinea. Pages 13–120 *in* L. Corbari, S. T. Ahyong, and T.-Y. Chan, editors. Deep-Sea Crustaceans from Papua New Guinea. Tropical Deep-Sea Benthos 31. Muséum national d'Histoire naturelle, Paris.

Mantelatto, F. L., M. Terossi, M. Negri, C. Raquel, R. Robles, T. Magalhães, A. F. Tamburus, N. Rossi, M. J. Miyazaki, F. L. Mantelatto, M. Terossi, M. Negri, C. Raquel, R. Robles, T. Magalhães, A. F. Tamburus, N. Rossi, F. L. Mantelatto, M. Terossi, M. Negri, R. C. Buranelli, R. R. Ã, T. Magalh, and A. F. Tamburus. 2017. DNA sequence database as a tool to identify decapod crustaceans on the São Paulo coastline Paulo coastline. Mitochondrial DNA Part A 0:1–11.

Pérez-Barros, P., M. E. D. Amato, N. V Guzmán, and G. A. Lovrich. 2008. Taxonomic status of two South American sympatric squat lobsters , *Munida gregaria* and *Munida subrugosa* (Crustacea: Decapoda: Galatheidae , challenged by DNA sequence information. Biological Journal of the Linnean Society 94:421–434.

Pérez-losada, M., C. G. Jara, G. Bond-Buckup, and K. A. Crandall. 2002. Phylogenetic relationships among the species of *Aegla* (Anomura: Aeglidae) freshwater crabs from Chile. Journal of Crustacean Biology 22:304–313.

Terrat, Y., E. Bonnivard, and D. Higuet. 2008. GalEa retrotransposons from galatheid squat

lobsters (Decapoda, Anomura) de W ne a new clade of Ty1/copia-like elements restricted to aquatic species. Molecular Genetics and Genomics 279:63–73.
